# Obesity and head and neck cancer risk: a mendelian randomization study

**DOI:** 10.1186/s12920-023-01634-4

**Published:** 2023-08-24

**Authors:** Lin Gui, Xiaohui He, Le Tang, Jiarui Yao, Jinping Pi

**Affiliations:** 1 Department of Medical Oncology, National Clinical Research Center for Cancer/Cancer Hospital, National Cancer Center, Chinese Academy of Medical Sciences & Peking Union Medical College, No. 17 Panjiayuan Nanli, Chaoyang District, Beijing, 100021 China; 2 Department of Medical Oncology, Beijing Chao yang District San huan Cancer Hospital, Beijing, 100122 China

**Keywords:** Mendelian randomization, Obesity, Head and neck cancer, BMI

## Abstract

**Background:**

Observational studies have reported controversial results on the association between obesity and head and neck cancer risk. This study aimed to perform a two-sample Mendelian randomization (MR) analysis to assess the causal association between obesity and head and neck cancer risk using publicly available genome-wide association studies (GWAS) summary statistics.

**Methods:**

Single-nucleotide polymorphisms (SNPs) for obesity [body mass index (BMI), waist-to-hip ratio (WHR), whole body fat mass, lean body mass, and trunk fat mass] and head and neck cancer (total head and neck cancer, oral cavity cancer, oropharyngeal cancer, and oral cavity and oropharyngeal cancer) were retrieved from published GWASs and used as genetic instrumental variables. Five methods including inverse-variance-weighted (IVW), weighted-median, MR–Egger, weighted mode, and MR-PRESSO were used to obtain reliable results, and odds ratio with 95% confidence interval (CI) were calculated. Tests for horizontal pleiotropy, heterogeneity, and sensitivity were performed separately.

**Results:**

Genetically predicted BMI was negatively associated with the risk of total head and neck cancer, which was significant in the IVW [OR (95%CI), 0.990 (0.984–0.996), *P* = 0.0005], weighted-median [OR (95%CI), 0.984 (0.975–0.993), *P* = 0.0009], and MR-PRESSO [OR (95%CI), 0.990 (0.984–0.995), *P* = 0.0004] analyses, but suggestive significant in the MR-Egger [OR (95%CI), 0.9980 (0.9968–0.9991), *P* < 0.001] and weighted mode [OR (95%CI), 0.9980 (0.9968–0.9991), *P* < 0.001] analyses. Similar, genetically predicted BMI adjust for smoking may also be negatively associated with the risk of total head and neck cancer (*P* < 0.05). Genetically predicted BMI may be negatively related to the risk of oral cavity cancer, oropharyngeal cancer, and oral cavity and oropharyngeal cancer (*P* < 0.05), but no causal association was observed for BMI adjust for smoking (*P* > 0.05). In addition, no causal associations were observed for other exposures and outcomes (all *P* > 0.05).

**Conclusion:**

This MR analysis supported the causal association of BMI-related obesity with decreased risk of total head and neck cancer. However, the effect estimates from the MR analysis were close to 1, suggesting a slight protective effect of BMI-related obesity on head and neck cancer risk.

## Background

Head and neck cancer is an umbrella term for a range of cancers including oral cavity, pharyngeal, nasopharyngeal, laryngeal, sinus, and salivary gland cancers [1]. In 2020, head and neck cancer were one of the most common cancers worldwide (1.43 million new cases and 0.51 million deaths), accounting for 7.4% of all cancers (19.3 million new cases) and 5.1% of all cancer deaths worldwide (10.0 million deaths) [2]. The prognosis and survival of head and neck cancer depend on tumor stage, site of involvement, and human papillomavirus (HPV) status [3]. Smoking and alcohol consumption, as well as high-risk types of HPV, are widely recognized as major risk factors for head and neck cancer [1]. Identifying potential factors associated with head and neck cancer plays an important role in disease prevention and prognosis management.

Obesity is associated with the development and progression of many cancers and is considered the second most common and modifiable cause of cancer development after smoking [4, 5]. Recently, several observational studies have reported an association between obesity and head and neck cancer risk [6–8]. Khanna et al. showed that being overweight and obese were significantly associated with a reduced risk of squamous cell carcinoma of the head and neck [6]. Chen et al. found that obesity was negatively associated with the risk of head and neck cancer [7]. Gaudet et al. reported that obesity was not associated with the incidence of head and neck cancer [8]. However, a meta-analysis demonstrated a positive association between BMI and head and neck cancer risk in non-smokers [9]. Inconsistent results among these observational studies may be influenced by confounding factors and reverse causality. Mendelian randomization (MR) studies, based on genetic epidemiology, have been widely used to explore the causal relationship between exposure and outcome [10, 11]. In contrast to traditional observational studies, MR studies use genetic variants associated with exposure to assess possible causality with outcomes and can reduce potential biases from confounding and reverse causality [12, 13].

This study aimed to assess the causal association between obesity exposure and head and neck cancer using genetic variants associated with obesity as non-confounding instruments.

## Methods

### Study design and data source

**Fig. 1:**
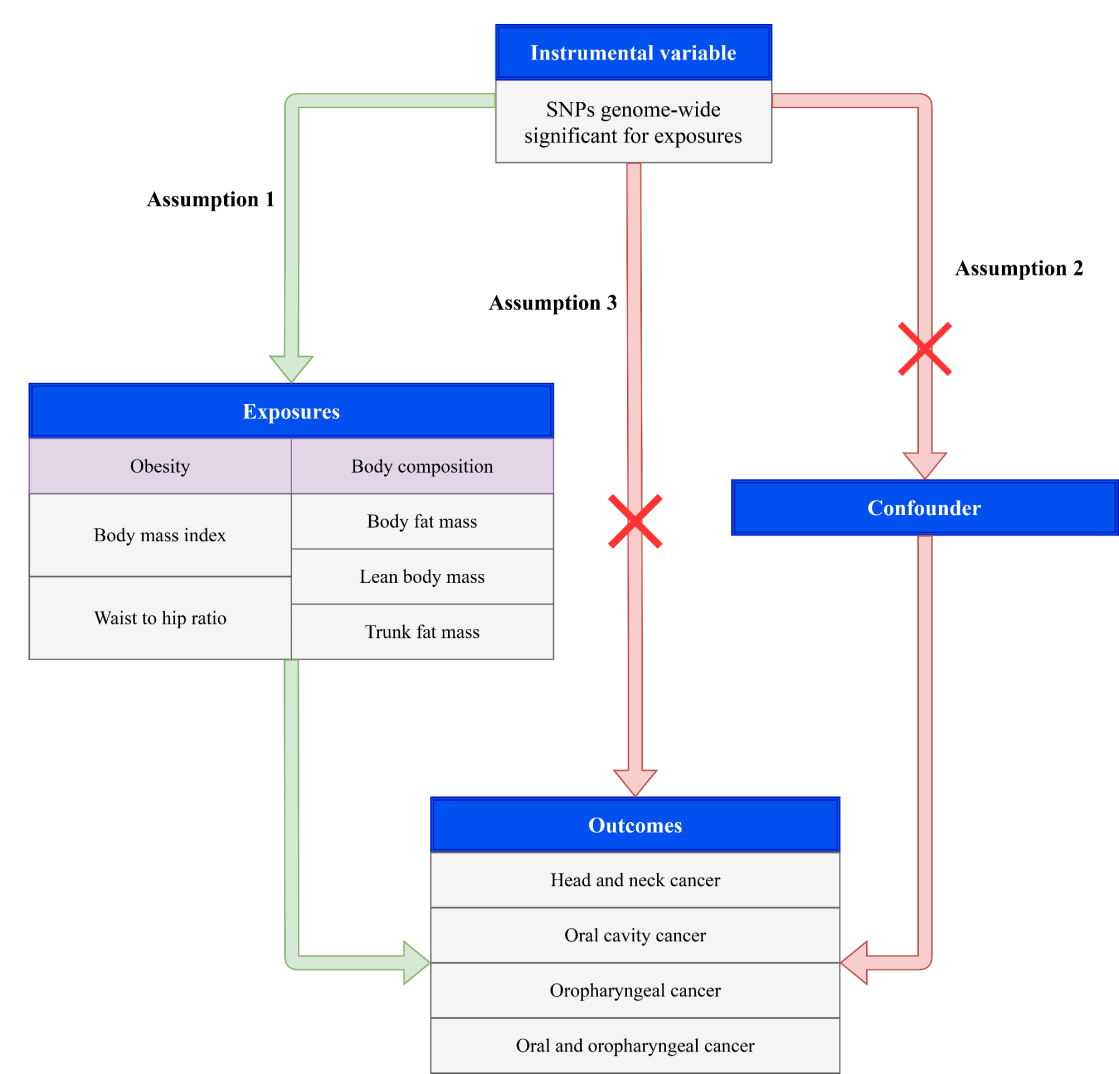
The Mendelian randomization study aims and assumptions. Assumption 1: genetic variants were associated with the exposure (relevance); assumption 2: genetic variates share no unmeasured cause with the outcome (independence); assumption 3: genetic variants do not affect outcome except through their potential effect on the exposure (exclusion restriction)

This study used two-sample MR to assess the relationship between obesity and head and neck cancer risk. All data used are publicly available and can be searched through the genome-wide association study (GWAS) Catalog (available at: https://www.ebi.ac.uk/gwas/) and the MRC Integrative Epidemiology Unit (IEU) database (available at: https://gwas.mrcieu.ac.uk/datasets/). Three assumptions were established include: (1) genetic variants were associated with the exposure (relevance); (2) genetic variates share no unmeasured cause with the outcome (independence); (3) genetic variants do not affect outcome except through their potential effect on the exposure (exclusion restriction) (Fig. 1). The requirement of ethical approval for this was waived by the Institutional Review Board of National Cancer Center/National Clinical Research Center for Cancer/Cancer Hospital, Chinese Academy of Medical Sciences & Peking Union Medical College, because the data was accessed from GWAS database (a publicly available database). The need for written informed consent was waived by the Institutional Review Board of National Cancer Center/National Clinical Research Center for Cancer/Cancer Hospital, Chinese Academy of Medical Sciences & Peking Union Medical College due to retrospective nature of the study. All methods were performed in accordance with the relevant guidelines and regulations.

### Instrumental variable selection

Single-nucleotide polymorphisms (SNPs) associated with exposure (obesity) and outcome (head and neck cancer) were summarized in Table 1. The variables included in exposure were body mass index (BMI), BMI adjusted for smoking, waist-to-hip ratio (WHR), WHR adjusted for BMI, WHR adjusted for BMI in non-smoker, WHR adjusted for BMI and smoking, whole body fat mass, lean body mass, and trunk fat mass. Instrumental variables for BMI were based on a meta-analysis of GWAS of 97 BMI-associated loci in 339,224 Europeans [14]. Instrumental variables for BMI adjusted for smoking, WHR adjusted for BMI in non-smoker, and WHR adjusted for BMI and smoking were obtained from a meta-analysis of GWAS on obesity in 241,258 Europeans [15]. Instrumental variables for WHR and WHR adjusted for BMI were derived from a meta-analysis of GWAS of 49 WHR-associated loci in 224,459 Europeans [16]. Instrumental variables for lean body mass were based on a meta-analysis of GWAS in 8,327 Europeans [17]. Instrumental variables for whole body fat mass and trunk fat mass were derived from the UK Biobank and obtained from the MRC IEU database. The variables included in the outcome were total head and neck cancer, oral cavity cancer, oropharyngeal cancer, and oral cavity and oropharyngeal cancer. Instrumental variables for total head and neck cancer, oral cavity cancer, oropharyngeal cancer, and oral cavity and oropharyngeal cancer were derived from the UK Biobank and obtained from the MRC IEU database. Specifically, 373,122 Europeans were included when total head and neck cancer was the outcome, including 1,106 head and neck cancer patients; 372,373 Europeans were included when oral cavity cancer was the outcome, including 357 oral cavity cancer patients; 372,510 Europeans were included when oropharyngeal cancer was the outcome, including 494 oropharyngeal cancer patients; and 372,855 Europeans were included when oral cavity and oropharyngeal cancer was the outcome, including 839 oral cavity and oropharyngeal cancer patients. For the selection of instrumental variables, a series of quality control steps were used to select eligible instrumental SNPs. First, SNPs with genome-wide significance (*P* < 5 × 10^− 8^) associated with exposure were selected. Second, the selected SNPs are not in linkage disequilibrium (LD) (r^2^ < 0.001, genetic distance = 10,000 kb). Third, the selected SNPs with minor allele frequency (MAF) < 0.01 were deleted. The F statistic and variance explained (R^2^) is also an indicator to assess the strength of the relationship between SNPs and exposure. If the F statistic of the SNPs-exposure association is > 10, the likelihood of weak instrumental variable bias is small [18].

**Table 1:** Summary of genetic variants associated with exposure (obesity) and outcome (head and neck cancer)

Variables	Source	Year	Searching	Sample size	Region	SNPs with significant (*P* < 5 × 10^− 8^)	SNPs without LD [r^2^, kb (0.01, 10,000)
Exposures							
BMI	GWAS-Catlog	2015	Locke et al.^14^	339,224	European	4,971	84
BMI adjust for smoking	GWAS-Catlog	2017	Justice et al.^15^	241,258	European	2,883	22
WHR	GIANT	2015	Shungin et al.^16^	224,459	European	506	31
WHR adjust for BMI	GWAS-Catlog	2015	Shungin et al.^16^	224,459	European	465	48
WHR adjust for BMI in non-smoker	GWAS-Catlog	2017	Justice et al.^15^	241,258	European	1,537	4
WHR adjust for BMI and smoking	GWAS-Catlog	2017	Justice et al.^15^	241,258	European	1,537	20
Whole body fat mass	MRC IEU (UK Biobank)	2018	GWAS ID: ukb-b-19,393	454,137	European	60,088	435
Lean body mass	GWAS-Catlog	2017	Medina-Gomez et al.^17^	8,327	European	308	47
Trunk fat mass	MRC IEU (UK Biobank)	2018	GWAS ID: ukb-b-20,044	454,588	European	61,670	422
Outcomes							
Head and neck cancer	MRC IEU (UK Biobank)	2021	GWAS ID: ieu-b-4912	373,122	European	NA	NA
Oral cavity cancer	MRC IEU (UK Biobank)	2021	GWAS ID: ieu-b-4961	372,373	European	NA	NA
Oropharyngeal cancer	MRC IEU (UK Biobank)	2021	GWAS ID: ieu-b-4968	372,510	European	NA	NA
Oral cavity and oropharyngeal cancer	MRC IEU (UK Biobank)	20,211	GWAS ID: ieu-b-4962	372,855	European	NA	NA

Note: SNP, single-nucleotide polymorphisms; BMI, body mass index; WHR, waist-to-hip ratio; GWAS, genome-wide association study; MRC IEU, MRC Integrative Epidemiology Unit database; NA, not applicable; LD, linkage disequilibrium

### Pleiotropy

Pleiotropy is the association of a genetic variant with multiple risk factors on different causal pathways. Horizontal pleiotropy of genes would violate assumption 2 and assumption 3 of the three assumptions of MR, and MR analysis should be performed on the premise of ensuring no horizontal pleiotropy. SNPs associated with confounders were excluded (assumption 2). SNPs directly related to the outcome without affecting outcome through exposure were excluded (assumption 3). MR-Egger Intercept test was used to test for horizontal pleiotropy, and the *P*-value for the intercept > 0.05 indicates no horizontal pleiotropic effects. MR-Egger is a weighted linear regression method based on the Instrument Strength Independent of Direct Effect (InSIDE) assumption which can give valid tests and consistent estimates of causal effects even if all instrumental variables are invalid [19].

### Statistical analysis

Two-sample MR analysis was performed using the “TwoSampleMR” package in R software. Exposure-SNP and outcome-SNP were harmonized using the “harmonise_data” function of the TwoSampleMR package so that variant effect estimates corresponded to the same allele. For each SNP in each exposure, individual MR effect estimates were calculated using the Wald method (SNP-exposure beta/SNP-outcome beta). Five methods including fixed-effects inverse-variance weighted (IVW), MR-Egger, weighted-median, weighted mode, and MR-PRESSO methods were used to analyze the association between obesity and head and neck cancer risk. The IVW method uses a meta-analysis approach to combine the Wald estimates for each SNP to obtain an overall estimate of the effect of obesity on head and neck cancer risk [20], which was the primary analysis used to generate causal effect estimates in the current study. The weighted-median method provides a robust and consistent estimate of the effect, even if nearly 50% of genetic variants were invalid instruments [21]. When most of the instrumental variables with similar causal estimates are valid, the weighted model method remains plausible even if some of the instrumental variables do not meet the requirements of the MR for causal inference [22]. The MR-PRESSO method detects horizontal pleiotropy and corrects for horizontal pleiotropy by removing outliers, as well as determining whether there is a substantial change in causal effects before and after removal of outliers [23]. However, the MR-PRESSO outlier test requires at least 50% of the genetic variation to be a valid tool and relies on the InSIDE assumption. Cochran’s Q test was utilized to evaluate the statistical heterogeneity among SNPs in the IVW method, and *P* < 0.05 was considered significant heterogeneity. The leave-one-out analyses were used to assess the reliance of an MR analysis on a particular SNP, and the symmetry directly observed in the plot indicates null pleiotropy. Furthermore, the bidirectional MR analysis was conducted to determine if there was a reverse causal association between exposure and outcome. The odds ratio (OR) and 95% confidence interval (CI) were calculated to evaluate the results of the MR analysis. For exposure BMI and BMI adjust after smoking, the OR values showed results for each increase of 5-units. *P*-value < 0.00139 = 0.05/36 (9 exposures and 4 outcomes) was considered statistically significant, whereas *P*-value between 0.00139 and 0.05 was considered suggestive significance in the IVW, weighted-median, MR-Egger, weighted mode, and MR-PRESSO analyses. All statistical analyses were performed by R software, version 4.1.1.

## Results

### Selection of instrument variables

Table 2 reports the number of SNPs and the results of SNP tests that were ultimately included in the analysis. After screening, 78 SNPs associated with BMI and 28 SNPs associated with WHR were retained when head and neck cancer was the outcome. There were 75 BMI-related SNPs and 29 WHR-related SNPs retained when oral cavity cancer was the outcome. There were 78 SNPs related to BMI and 28 SNPs related to WHR retained when oropharyngeal cancer was the outcome. There were 69 BMI-related SNPs and 27 WHR-related SNPs retained when oral cavity and oropharyngeal cancer was the outcome. The MR-Egger analysis showed no horizontal pleiotropy for all retained SNPs (all *P* > 0.05). The F statistics associated with each SNP-exposure was much greater than 10, indicating that the possibility of weak instrumental variable bias was small. Furthermore, the results of the heterogeneity test showed that the selected SNPs were not heterogeneous (all *P* > 0.05).

**Table 2:** The number of SNPs used for analysis and their pleiotropy, strength, and heterogeneity tests

Outcome	Exposure	Uncorrelated pleiotropy	Correlated pleiotropy	Strength	Heterogeneity	
		Number of SNPs (non-MAF)	MR-Egger (Intercept, *P*)	F-statistic, R^2^	MR-Egger (Q statistic, *P*)	IVW (Q statistic, *P*)
Head and neck cancer	BMI	78	-0.0000, 0.8500	25.035, 0.6	69.4002, 0.6905	69.4362, 0.7179
	BMI adjust for smoking	20	0.0001, 0.4909	20.518, 0.2	21.2557, 0.2667	21.8396, 0.2923
	WHR	28	0.0000, 0.7136	19.208, 0.2	23.4464, 0.6076	23.5842, 0.6533
	WHR adjust for BMI	44	0.0000, 0.7505	21.091, 0.4	39.0745, 0.6001	39.1769, 0.6378
	WHR adjust for BMI in non-smoker	4	0.0002, 0.6536	29.379, 0.1	0.9857, 0.6109	1.2584, 0.7390
	WHR adjust for BMI and smoking	19	0.0001, 0.4517	24.229, 0.2	16.7629, 0.4705	17.3562, 0.4988
	Whole body fat mass	399	0.0000, 0.337	24.037, 2.1	388.4259, 0.6113	389.3501, 0.6122
	Lean body mass	44	-0.0001, 0.1205	6.498, 3.4	30.0598, 0.9158	32.5710, 0.8765
	Trunk fat mass	386	0.0000, 0.1656	24.154, 2.1	395.6752, 0.3296	397.6635, 0.3172
Oral cavity cancer	BMI	75	0.0000, 0.5877	25.654, 0.6	57.6927, 0.9052	57.9892, 0.9145
	BMI adjust for smoking	20	0.0001, 0.1563	21.181, 0.2	17.8527, 0.4654	20.0416, 0.3921
	WHR	29	0.0001, 0.4294	19.039, 0.2	28.7347, 0.3739	29.4196, 0.3915
	WHR adjust for BMI	44	0.0001, 0.2917	21.155, 0.4	31.2901, 0.8871	32.4302, 0.8802
	WHR adjust for BMI in non-smoker	4	0.0003, 0.3651	29.379, 0.1	3.0021, 0.2229	5.0293, 0.1697
	WHR adjust for BMI and smoking	19	0.0001, 0.1542	24.229, 0.2	12.9371, 0.7404	15.1609, 0.6509
	Whole body fat mass	386	-0.0000, 0.9999	24.785, 2.1	342.3921, 0.9376	342.3921, 0.9420
	Lean body mass	43	-0.0000, 0.5551	6.558, 3.4	29.6563, 0.9059	30.0104, 0.9168
	Trunk fat mass	376	0.0000, 0.5103	24.92, 2.1	345.6535, 0.8507	346.0878, 0.8553
Oropharyngeal cancer	BMI	78	-0.0000, 0.9903	24.704, 0.6	68.3080, 0.7230	68.3082, 0.7500
	BMI adjust for smoking	19	0.0000, 0.7971	20.928, 0.2	13.5383, 0.6995	13.6066, 0.7544
	WHR	28	0.0000, 0.9531	18.955, 0.2	18.9115, 0.8401	18.9151, 0.8731
	WHR adjust for BMI	44	0.0000, 0.5067	21.135, 0.4	42.8039, 0.4365	43.2611, 0.4602
	WHR adjust for BMI in non-smoker	7	0.0000, 0.9478	29.39, 0.1	4.6050, 0.4660	4.6097, 0.5948
	WHR adjust for BMI and smoking	19	0.0001, 0.3156	24.229, 0.2	19.0014, 0.3285	20.1968, 0.3218
	Whole body fat mass	396	0.0000, 0.3228	24.103, 2.1	353.4590, 0.9296	354.4391, 0.9295
	Lean body mass	44	-0.0001, 0.0791	6.446, 3.4	35.4305, 0.7531	38.6684, 0.6595
	Trunk fat mass	383	0.0000, 0.0709	24.239, 2.0	363.6304, 0.7307	366.9096, 0.7016
Oral cavity and oropharyngeal cancer	BMI	77	-0.0000, 0.8766	25.265, 0.6	67.8745, 0.7076	67.8987, 0.7348
	BMI adjust for smoking	20	0.0000, 0.9455	20.518, 0.2	24.1838, 0.1491	24.1903, 0.1890
	WHR	26	0.0001, 0.5159	19.709, 0.2	22.4942, 0.5498	22.9290, 0.5817
	WHR adjust for BMI	41	0.0001, 0.1895	20.407, 0.4	41.0630, 0.3802	42.9405, 0.3463
	WHR adjust for BMI in non-smoker	6	0.0001, 0.6145	29.59, 0.1	2.3738, 0.6674	2.6711, 0.7505
	WHR adjust for BMI and smoking	16	0.0003, 0.1228	24.969, 0.2	10.9424, 0.6906	13.6399, 0.5530
	Whole body fat mass	396	0.0000, 0.4551	24.07, 2.1	351.0370, 0.9413	351.5961, 0.9430
	Lean body mass	43	-0.0001, 0.1773	6.564, 3.4	36.5767, 0.6674	38.4609, 0.6271
	Trunk fat mass	383	0.0000, 0.0709	24.239, 2.0	363.6304, 0.7307	366.9096, 0.7016

Note: SNP, single-nucleotide polymorphisms; BMI, body mass index; WHR, waist-to-hip ratio; MAF, minor allele frequency; MR, Mendelian randomization; IVW, inverse-variance weighted

### Two-sample MR analysis for causal association of obesity with head and neck cancer

Table 3 shows the causal association between obesity and head and neck cancer risk. The results demonstrated that genetically predicted BMI was negatively associated with the risk of total head and neck cancer, which was significant in the IVW [OR (95%CI), 0.990 (0.984–0.996), *P* = 0.0005], weighted-median [OR (95%CI), 0.984 (0.975–0.993), *P* = 0.0009], and MR-PRESSO [OR (95%CI), 0.990 (0.984–0.995), *P* = 0.0004] analyses, but suggestive significant in the MR Egger [OR (95%CI), 0.9980 (0.9968–0.9991), *P* < 0.001] and weighted mode [OR (95%CI), 0.9980 (0.9968–0.9991), *P* < 0.001] analyses. For oral cavity cancer, genetically predicted BMI may be negatively related to the risk of oral cavity cancer, which was suggestive significant in the weighted-median [OR (95%CI), 0.992 (0.987–0.997), *P* = 0.0033], weighted mode [OR (95%CI), 0.991 (0.983–0.998), *P* = 0.0154], and MR-PRESSO [OR (95%CI), 0.997 (0.994-1.000), *P* = 0.0347] analyses. For oropharyngeal cancer, genetically predicted BMI may be negatively related to the risk of oropharyngeal cancer, which was suggestive significant in the IVW [OR (95%CI), 0.996 (0.992–0.999), *P* = 0.0287] and MR-PRESSO [OR (95%CI), 0.996 (0.992–0.999), *P* = 0.0228] analyses. For oral and oropharyngeal cancer, genetically predicted BMI may be negatively related to the risk of oral and oropharyngeal cancer, which was suggestive significant in the IVW [OR (95%CI), 0.993 (0.988–0.998), *P* = 0.0037], weighted-median [OR (95%CI), 0.991 (0.982–0.999), *P* = 0.0325], weighted mode [OR (95%CI), 0.988 (0.978–0.999), *P* = 0.0302], and MR-PRESSO [OR (95%CI), 0.993 (0.988–0.997), *P* = 0.003] analyses.

**Table 3:** Causal association between obesity and head and neck cancer risk

Exposure	Methods	Head and neck cancer		Oral cavity cancer		Oropharyngeal cancer		Oral and oropharyngeal cancer	
		OR (95%CI)	*P*	OR (95%CI)	*P*	OR (95%CI)	*P*	OR (95%CI)	*P*
BMI	MR Egger	**0.987 (0.974-1.000)**	**0.018**	0.993 (0.985–1.001)	0.051	0.998 (0.989–1.006)	0.31	0.990 (0.978–1.002)	0.051
	Weighted median	**0.984 (0.975–0.993)**	**0.0009**	**0.992 (0.987–0.997)**	**0.0033**	0.998 (0.992–1.005)	0.5625	**0.991 (0.982–0.999)**	**0.0325**
	Inverse variance weighted	**0.990 (0.984–0.996)**	**0.0005**	0.997 (0.993-1.000)	0.0568	**0.996 (0.992–0.999)**	**0.0287**	**0.993 (0.988–0.998)**	**0.0037**
	Weighted mode	**0.985 (0.973–0.997)**	**0.0176**	**0.991 (0.983–0.998)**	**0.0154**	0.997 (0.990–1.005)	0.4603	**0.988 (0.978–0.999)**	**0.0302**
	MR-PRESSO	**0.990 (0.984–0.995)**	**0.0004**	**0.997 (0.994-1.000)**	**0.0347**	**0.996 (0.992–0.999)**	**0.0228**	**0.993 (0.988–0.997)**	**0.003**
BMI adjust for smoking	MR Egger	**0.980 (0.962–0.999)**	**0.016**	0.991 (0.981–1.001)	0.051	0.998 (0.985–1.011)	0.381	0.988 (0.972–1.004)	0.071
	Weighted median	**0.983 (0.969–0.998)**	**0.0215**	0.992 (0.984-1.000)	0.0531	0.998 (0.988–1.007)	0.6113	0.989 (0.977–1.001)	0.0804
	Inverse variance weighted	**0.988 (0.978–0.999)**	**0.0314**	0.997 (0.991–1.003)	0.2826	0.997 (0.991–1.004)	0.4005	0.990 (0.981-1.000)	0.0525
	Weighted mode	**0.981 (0.967–0.995)**	**0.0158**	0.991 (0.982-1.000)	0.0556	0.998 (0.988–1.008)	0.6851	0.988 (0.974–1.001)	0.0889
	MR-PRESSO	**0.988 (0.978–0.999)**	**0.0445**	0.997 (0.991–1.003)	0.2961	0.997 (0.991–1.003)	0.3464	0.990 (0.981-1.000)	0.0675
WHR	MR Egger	0.997 (0.990–1.005)	0.253	1.000 (0.996–1.004)	0.487	1.000 (0.995–1.005)	0.444	0.999 (0.992–1.006)	0.382
	Weighted median	0.998 (0.995–1.001)	0.1739	0.999 (0.998–1.001)	0.4755	0.999 (0.997–1.001)	0.5512	**0.997 (0.994-1.000)**	**0.0265**
	Inverse variance weighted	**0.998 (0.996-1.000)**	**0.0337**	0.999 (0.998-1.000)	0.0973	1.000 (0.999–1.001)	0.9132	0.998 (0.996-1.000)	0.081
	Weighted mode	0.996 (0.991–1.002)	0.1841	1.001 (0.997–1.005)	0.662	1.000 (0.996–1.003)	0.7962	0.996 (0.992–1.001)	0.1182
	MR-PRESSO	**0.998 (0.996-1.000)**	**0.0313**	0.999 (0.998-1.000)	0.1085	1.000 (0.999–1.001)	0.8973	0.998 (0.997-1.000)	0.0804
WHR adjust for BMI	MR Egger	0.997 (0.992–1.002)	0.107	0.999 (0.996–1.002)	0.246	0.998 (0.995–1.002)	0.149	0.997 (0.992–1.002)	0.105
	Weighted median	0.999 (0.996–1.001)	0.2271	0.999 (0.998–1.001)	0.3317	0.999 (0.998–1.001)	0.3862	0.998 (0.996-1.000)	0.1031
	Inverse variance weighted	0.999 (0.997–1.001)	0.2527	1.000 (0.999–1.001)	0.8027	1.000 (0.999–1.001)	0.5291	0.999 (0.998–1.001)	0.4382
	Weighted mode	0.997 (0.993–1.001)	0.2068	0.999 (0.997–1.001)	0.4361	0.999 (0.996–1.002)	0.4694	0.997 (0.994–1.001)	0.1596
	MR-PRESSO	0.999 (0.997–1.001)	0.2436	1.000 (0.999–1.001)	0.7749	1.000 (0.999–1.001)	0.5324	0.999 (0.998–1.001)	0.4428
WHR adjust for BMI in non-smoker	MR Egger	0.993 (0.973–1.014)	0.244	0.993 (0.981–1.006)	0.144	0.997 (0.984–1.011)	0.347	0.993 (0.976–1.011)	0.205
	Weighted median	0.996 (0.991–1.002)	0.2213	0.998 (0.994–1.001)	0.2221	0.999 (0.995–1.003)	0.705	0.997 (0.991–1.002)	0.2196
	Inverse variance weighted	0.997 (0.992–1.002)	0.2011	0.998 (0.996–1.001)	0.2326	0.999 (0.996–1.002)	0.3671	0.997 (0.993–1.001)	0.1689
	Weighted mode	0.996 (0.988–1.004)	0.3708	0.996 (0.991–1.002)	0.2556	0.999 (0.993–1.004)	0.6512	0.997 (0.990–1.004)	0.3946
	MR-PRESSO	0.997 (0.995-1.000)	0.057	0.998 (0.996–1.001)	0.2754	0.999 (0.996–1.001)	0.3431	0.997 (0.994-1.000)	0.1185
WHR adjust for BMI and smoking	MR Egger	0.997 (0.986–1.009)	0.301	0.997 (0.991–1.004)	0.219	0.997 (0.990–1.005)	0.225	0.996 (0.986–1.006)	0.224
	Weighted median	0.998 (0.995–1.001)	0.1999	1.000 (0.998–1.001)	0.6475	1.000 (0.998–1.002)	0.9734	0.998 (0.995–1.001)	0.1753
	Inverse variance weighted	0.999 (0.997–1.001)	0.4494	1.000 (0.998–1.001)	0.6971	1.000 (0.998–1.001)	0.6187	0.999 (0.997–1.001)	0.38
	Weighted mode	0.998 (0.992–1.003)	0.3614	1.000 (0.997–1.002)	0.7734	1.000 (0.996–1.004)	0.9631	0.997 (0.993–1.002)	0.2348
	MR-PRESSO	0.999 (0.997–1.001)	0.4511	1.000 (0.998–1.001)	0.6995	1.000 (0.998–1.001)	0.6248	0.999 (0.997–1.001)	0.3718
Whole body fat mass	MR Egger	0.998 (0.996-1.000)	0.052	1.000 (0.998–1.001)	0.256	0.999 (0.998–1.001)	0.127	0.999 (0.997-1.000)	0.055
	Weighted median	0.999 (0.998-1.000)	0.1006	0.999 (0.999-1.000)	0.1419	1.000 (0.999–1.001)	0.7185	0.999 (0.998-1.000)	0.1657
	Inverse variance weighted	1.000 (0.999–1.001)	0.4998	1.000 (0.999-1.000)	0.2653	1.000 (1.000-1.001)	0.846	1.000 (0.999-1.000)	0.2323
	Weighted mode	0.998 (0.995–1.001)	0.1274	0.998 (0.996-1.000)	0.0304	1.000 (0.998–1.001)	0.7225	0.998 (0.995–1.001)	0.1276
	MR-PRESSO	1.000 (0.999–1.001)	0.4955	1.000 (0.999-1.000)	0.2397	1.000 (1.000-1.001)	0.8377	1.000 (0.999-1.000)	0.2062
Lean body mass	MR Egger	0.999 (0.997–1.002)	0.331	1.000 (0.998–1.001)	0.328	1.000 (0.999–1.002)	0.331	1.000 (0.998–1.002)	0.462
	Weighted median	1.000 (0.999–1.001)	0.9473	1.000 (0.999-1.000)	0.764	1.000 (1.000-1.001)	0.4834	1.000 (0.999–1.001)	0.8908
	Inverse variance weighted	1.000 (0.999–1.001)	0.8881	1.000 (1.000–1.000)	0.6678	1.000 (1.000-1.001)	0.5034	1.000 (1.000-1.001)	0.9009
	Weighted mode	1.000 (0.998–1.002)	0.9713	1.000 (0.999–1.001)	0.8471	1.000 (0.999–1.001)	0.7963	1.000 (0.998–1.002)	0.8159
	MR-PRESSO	1.000 (0.999-1.000)	0.8723	1.000 (1.000–1.000)	0.6143	1.000 (1.000-1.001)	0.4843	1.000 (1.000-1.001)	0.8971
Trunk fat mass	MR Egger	0.999 (0.996–1.001)	0.11	1.000 (0.998–1.001)	0.245	0.999 (0.998–1.001)	0.178	0.999 (0.997-1.000)	0.061
	Weighted median	0.999 (0.998–1.001)	0.2767	1.000 (0.999-1.000)	0.4351	1.000 (0.999–1.001)	0.4721	0.999 (0.998-1.000)	0.1263
	Inverse variance weighted	1.000 (0.999–1.001)	0.5994	1.000 (1.000–1.000)	0.8589	1.000 (1.000-1.001)	0.6184	1.000 (0.999–1.001)	0.7999
	Weighted mode	0.998 (0.995–1.001)	0.2085	0.999 (0.997-1.000)	0.1413	0.999 (0.998–1.001)	0.5802	0.998 (0.995-1.000)	0.1097
	MR-PRESSO	1.000 (0.999–1.001)	0.5997	1.000 (1.000–1.000)	0.8535	1.000 (1.000-1.001)	0.6108	1.000 (0.999–1.001)	0.796

Note: BMI, body mass index; WHR, waist-to-hip ratio; MR, Mendelian randomization; OR, odds ratio; 95%CI, 95% confidence interval

**Fig. 2:**
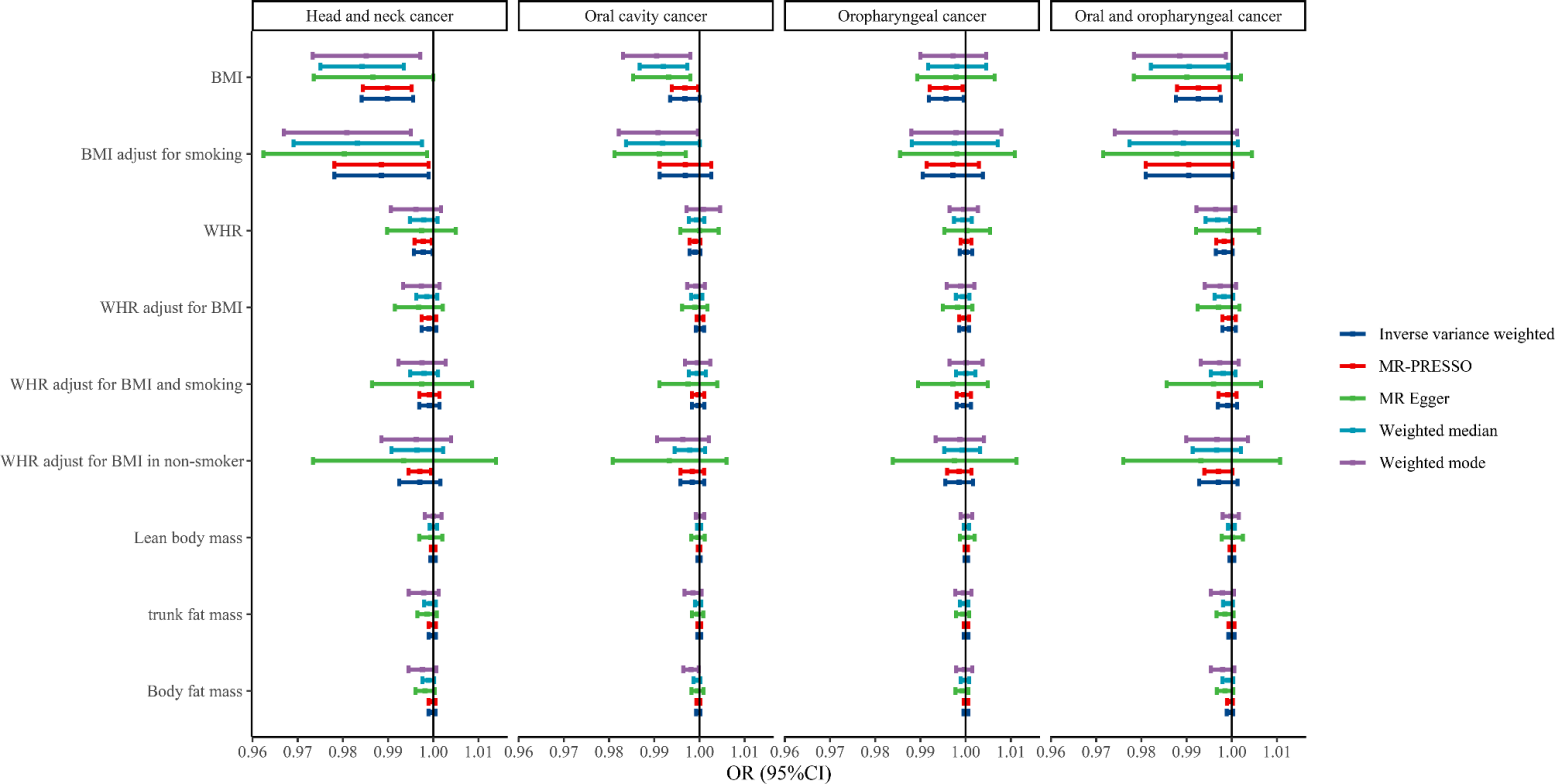
Forest plots of the causal link between obesity and head and neck cancer risk. BMI, body mass index; WHR, waist-to-hip ratio; OR, odds ratio; 95%CI, 95% confidence interval

For exposure BMI adjust for smoking, genetically predicted BMI adjust for smoking may be negatively associated with the risk of head and neck cancer, which was suggestive significant in the IVW [OR (95%CI), 0.988 (0.978–0.999), *P* = 0.0314], MR Egger [OR (95%CI), 0.980 (0.962–0.999), *P* = 0.016], weighted-median [OR (95%CI), 0.983 (0.969–0.998), *P* = 0.0215], weighted mode [OR (95%CI), 0.981 (0.967–0.995), *P* = 0.0158], and MR-PRESSO [OR (95%CI), 0.988 (0.978–0.999), *P* = 0.0445] analyses. However, no causal associations were observed between BMI adjust for smoking and oral cavity cancer and oropharyngeal cancer and oral and oropharyngeal cancer (all *P* > 0.05). For exposure WHR, genetically predicted WHR may be negatively related to the risk of head and neck cancer, which was suggestive significant in the IVW [OR (95%CI), 0.998 (0.996-1.000), *P* = 0.0337] and MR-PRESSO [OR (95%CI), 0.998 (0.996-1.000), *P* = 0.0313] analyses. In addition, no causal associations between other exposures and head and neck cancer risk were observed (all *P* > 0.05). Figure 2 presents the forest plots of the causal link between obesity and head and neck cancer risk.

**Fig. 3:**
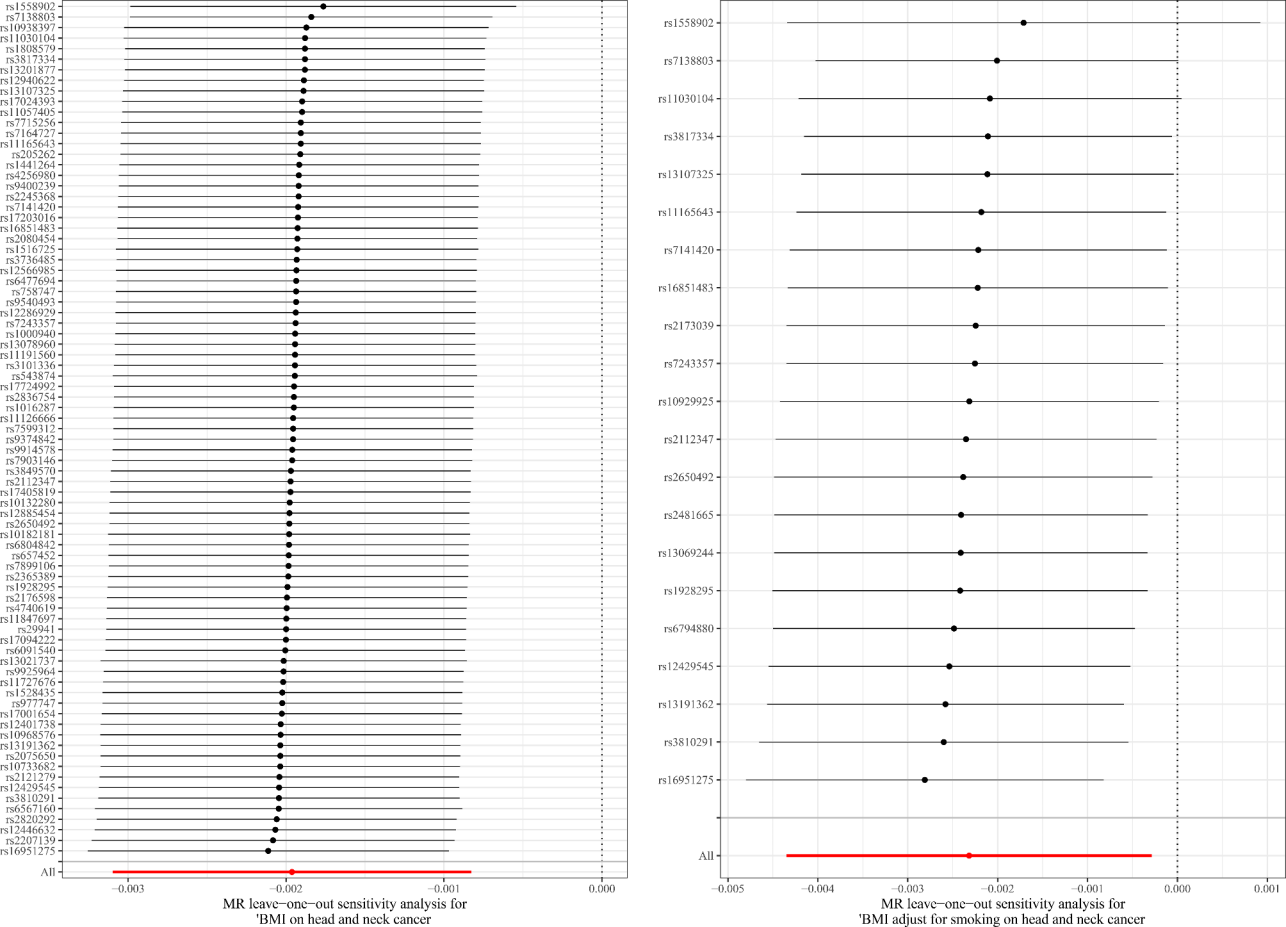
The leave-one-out analysis to assess the reliance of an MR analysis on a particular SNP. The symmetry directly observed in the plot indicates null pleiotropy. MR, Mendelian randomization; SNP, single-nucleotide polymorphisms; BMI, body mass index

The leave-one-out sensitivity analysis demonstrated that the relationship between obesity and head and neck cancer was not caused by any individual SNP (Fig. 3). Moreover, the bidirectional MR analysis showed that there was not a reverse causal association between BMI-related obesity and head and neck cancer [IVW: β (95%CI), 0.521 (-1.692, 2.734), *P* = 0.645; MR-Egger: β (95%CI), -0.633 (-5.873, 4.607), *P* = 0.820; weighted-median method: β (95%CI), 1.064 (-1.935, 4.064), *P* = 0.487].

## Discussion

This study used summary statistics from published GWAS for a two-sample MR analysis to explore the causal association between obesity and head and neck cancer risk. The results demonstrated that genetically predicted BMI-related obesity was negatively associated with the risk of total head and neck cancer, while no causal association was found between WHR-related obesity and total head and neck cancer risk.

Several published observational studies have reported inconsistent results regarding obesity and head and neck cancer [7–9]. Two cohort studies have reported that no statistically significant association was observed between obesity and head and neck cancer incidence [8, 24]. However, some studies have shown that a high BMI is associated with a lower risk of head and neck cancer [7, 25, 26]. Etemadi et al. reported that the risk of head and neck cancer was negatively associated with BMI among current smokers [26]. A meta-analysis of cohort studies showed that WHR was positively associated with the risk of head and neck cancer regardless of smoking, while a positive association with BMI was found only among never smokers [9]. To address the controversy between these results, this study used MR analysis to investigate the causal association between obesity and head and neck cancer risk. Our results found that BMI-related obesity was negatively associated with the risk of total head and neck cancer, and this association may be persisted after adjusting for smoking. However, a recent MR study by Gormley et al. showed no causal association between BMI and oral and oropharyngeal cancer risk [27]. Our results demonstrated that BMI may be negatively related to the risk of oropharyngeal cancer, but no causal association was found between BMI adjust for smoking and the risk of oropharyngeal cancer. It may be that smoking is an important confounder influencing the risk of head and neck cancer, as postulated by Gormley et al. However, both BMI and BMI adjust for smoking were causally associated with total head and neck cancer risk, suggesting that there may be a causal association between BMI and the risk of a particular type of head and neck cancer. In addition, the OR value of our MR analysis results was close to 1, which suggested that although there was a causal association between obesity and total head and neck cancer risk, obesity may not play a major role in the development of head and neck cancer. For specific types of head and neck cancer, the MR study by Fussey et al. found that there was not a causal association between obesity and benign nodular thyroid disease or thyroid cancer [28]. Since head and neck cancer includes the oral cavity, pharyngeal, nasopharyngeal, laryngeal, sinus and salivary gland cancers, the causal association between obesity and a particular type of head and neck cancer needs to be explored in subsequent studies.

The mechanism by which obesity reduces the risk of head and neck cancer remains unclear. A systematic review summarized the potential mechanisms between obesity and head and neck cancer [25]. Many substances associated with obesity such as fatty acid synthase, fatty acid binding protein, phospholipase A2, and adipokines may be involved in the development of head and neck cancer [25]. Adipokines are hormones secreted by adipose tissue that can regulate inflammatory and metabolic processes and influence cell growth and proliferation [29]. Adipokines associate obesity with low-grade chronic inflammation [30]. This low-grade chronic inflammation contributes to the formation of a tumor microenvironment that affects cell plasticity through epithelial-mesenchymal transition, dedifferentiation, immune cell polarization, reactive oxygen species, and cytokines [31, 32]. The association between dysregulation of adipokine and adipokine receptor expression and head and neck cancer has been demonstrated [33]. Pathways involved in these processes include Janus kinase/signal sensor and transcriptional activator (JAK/STAT pathway), Phosphatidylinositol kinase (PI3/Akt/mTOR), and Peroxisome proliferator activator receptor (PPAR) [33]. Leptin and adiponectin are the most studied adipokines. Obese people have higher levels of leptin than people of normal weight [34]. A decrease in circulating leptin levels can be detected in patients with cancer, including oral squamous cell carcinoma [35]. Decreased adiponectin levels were observed in the course of obesity and adipose tissue malnutrition [36]. Moreover, other potential factors such as HPV may also influence the association between obesity and head and neck cancer. Tan et al. found a negative association between obesity and the risk of head and neck squamous cell carcinoma in HPV seronegative cases, but not in HPV seropositive cases [37].

This study first investigated the causal association between obesity and head and neck cancer risk. This study may provide genetic evidence for the controversial results between obesity and head and neck cancer risk. However, several limitations of this study should be considered. First, although our study found the causal association between BMI-related obesity and head and neck cancer risk, we were unable to identified the specific type of head and neck cancer due to data limitations. Second, the study population included in the MR analysis was of European descent, and whether the results are representative of the entire population remains to be verified. Third, there should not be overlapping participants for exposure and outcome in a two-sample MR study, while the extent of sample overlap in the present study is unknown. However, the use of a strong instrument (e.g., F statistic > 10) in this study minimizes the potential bias of sample overlap.

## Conclusions

This two-sample MR study found that genetically predicted BMI-related obesity was negatively associated with the risk of total head and neck cancer. These findings provide new evidence for the etiology of head and neck cancer, although obesity may not be a major factor. Future studies could focus on the causal link between obesity and different types of head and neck cancer.

## Data Availability

The datasets generated during and/or analyzed during the current study are available in the GWAS Catalog, https://www.ebi.ac.uk/gwas/.

## List of abbreviations

HPV: Human papillomavirus
GWAS: Genome-wide association study
IEU: Integrative Epidemiology Unit
SNPs: Single-nucleotide polymorphisms
BMI: Body mass index
WHR: Waist-to-hip ratio
InSIDE: Instrument Strength Independent of Direct Effect
IVW: Inverse-variance weighted
